# Influence of Catheter Type and Tenaculum Use on Intrauterine Insemination Outcome

**DOI:** 10.22074/ijfs.2020.6161

**Published:** 2020-10-12

**Authors:** Pinar Gulsen Coban, Ayla Sargin Oruc, Meryem Kuru Pekcan, Hasan Ali Inal, Necati Hancerliogullari, Nafiye Yilmaz

**Affiliations:** 1 Department of Gynaecology and Obstetrics, Numune Education and Research Hospital, Ankara,Turkey; 2 Department of Gynaecology and Obstetrics, Ankara Guven Hospital, Ankara, Turkey; 3 Department of Gynaecology and Obstetrics, Zekai Tahir Burak Women’s Health and Research Hospital, Ankara, Turkey; 4 Department of Gynaecology and Obstetrics, Konya Education and Research Hospital, Konya, Turkey

**Keywords:** Catheter, Clinical Pregnancy Rate, Intrauterine Insemination, Live Birth Rate, Tenaculum

## Abstract

**Background:**

We investigated the impact of the choice of catheter type and tenaculum use on pregnancy related out- comes in intrauterine insemination (IUI) treatments.

**Materials and Methods:**

A total of 338 consecutive IUI cycles were assessed in this retrospective study. Participants were divided according to the insemination technique - soft catheter (group 1; n=175), firm catheter (group 2; n=100), or tenaculum (group 3; n=63). Clinical, laboratory, semen parameters and pregnancy related outcomes were compared.

**Results:**

Demographic characteristics and laboratory parameters were similar between the groups (P>0.05). The clini- cal pregnancy rate (CPR) was significantly higher in the firm catheter (19%, 19/100) and tenaculum (31.7%, 20/63) groups compared to the soft catheter group (5.1%, 9/175, P<0.001). There were no significant differences between the groups in live birth and miscarriage rates per clinical pregnancy (P>0.05).

**Conclusion:**

Our findings indicate that the use of a firm catheter or tenaculum for IUI might result in a higher CPR, but might not have a considerable effect on the live birth rate (LBR). Further prospective randomized studies are required to determine the long-term effects of the catheter type or tenaculum use on IUI success.

## Introduction

Intrauterine insemination (IUI) is an effective and widely used treatment that is mainly recommended for male factor, minimal and mild endometriosis, cervical factor or unexplained infertility cases. The term unexplained infertility includes infertile pairs whom ovulatory function, tubal passage and semen analysis are normal. The procedure involves the direct delivery of washed spermatozoa in order to bypass the cervix and increase the sperm volume at the site of fertilisation (1-4).

In the literature, the pregnancy rate reported in IUI cycles varies widely from 4-40% (5, 6). This great variation might be related to female age, type and duration of infertility, sperm parameters and technical aspects (7, 8). Under the heading of technical aspects, in particular, the catheter type can possibly influence pregnancy outcomes for IUI (9). In many recent in vitro fertilisation (IVF) studies, the consistency of the embryo transfer (ET) catheter has been determined to be a considerable factor in the success of ET, whereas the influence of catheter type in IUI is still controversial(10).

In a meta-analysis of 1871 IUI cycles, it was reported that endometrial scratch injury was associated with higher clinical pregnancy and ongoing pregnancy rates (11). The authors suggested that the local endometrial trauma and subsequent acute inflammatory process might have prompted decidualization and improved the implantation rate. On the other hand, Balci et al. reported that the immediate uterine contractions induced by tenaculum application to the cervix during IUI might enhance sperm transport to the ampulla and result in a higher pregnancy rate (12). In this study, we aimed to investigate whether firm catheter introduction or tenaculum use for IUI might affect pregnancy related outcomes through local endometrial injury, induced myometrial contractions, or in via other means as suggested above.

## Materials and Methods

This retrospective study was conducted on a total of 338 IUI cycles carried out at the Department of Obstetrics and Gynaecology, Zekai Tahir Burak Women’s Health Education and Research Hospital, Ankara, Turkey between 2015 and 2017. Written informed consent was obtained from the participants for future use. The patients were assigned to three groups - IUI performed with a soft catheter (group1, n=175); firm catheter (group 2, n=100); or with the assistance of a tenaculum to ease the introduction (group 3, n=63). The Ethics Committee of Zekai Tahir Burak Women’s Health Education and Research Hospital, Ankara, Turkey approved this study (reference number: 2017/20), which was conducted in accordance with the Declaration of Helsinki 2013 Brazil version (20796219-724.087).

Inclusion criteria for IUI consisted of unexplained infertility with a minimum duration of one year, age under 35 years, normal uterine cavity, at least one patent tube, basal follicle stimulating hormone (FSH) <10 mIU/mL, no history of gynaecologic surgery and at least 5 million motile spermatozoa for the male partner. The first and subsequent cycles were admitted to the study. Exclusion criteria were diminished ovarian reserve and male infertility.

Ovarian stimulation was achieved by recombinant FSH (recFSH; follitropin alfa, Gonal-F, Serono, Turkey, Istanbul; follitropin beta Puregon, Organon, Turkey) and human menopausal gonadotropin (hMG; Ferring, Turkey) based on the patient’s historical and clinical factors. recFSH andhMG were administered in a low-dose step up stimulation protocol that began on the second day of the menstrual cycle. Ovarian response was recorded through ultrasound examination of antral follicles and by determination of serum oestradiol (E2) levels. Ovulation was triggered by human chorionic gonadotropin (hCG) (u-hCG, Pregnyl, Organon,Turkey; rec-hCG, Ovitrelle, Serono, Turkey) when one or two follicles reached a diameter of ≥18 mm.Finally, IUI was carried out after 36 hours of hCG administration.

Semen was collected by masturbation after 3-5 days of sexual abstinence and a few hours prior to the scheduled insemination time. The spermatozoa were washed free from the seminal liquid and prepared for insemination by the swim-up technique. The difficulty of the insemination was determined with respect to the comments of two physicians with the same techniques. For the initial attempt to cannulate the cervix, a soft catheter (Allwin Medical Devices, CA, USA) was preferred; thereafter, due to the difficulty degree of introduction, a firm catheter (Technocath Medical Scientifics, Ankara, Turkey) or tenaculum were used for the insemination. Finally, the sperm sample (0.5-1 mL) was slowly injected through the catheter into the uterine cavity.

Approximately two weeks after insemination, all participants underwent pregnancy tests. The endpoints of the study were the clinical pregnancy rate (CPR), which was defined as evidence of a gestational sac after more than six weeks gestation confirmed by ultrasound and the live birth rate (LBR), which was defined as the delivery of a live foetus after 20 weeks of gestational age.

### Statistical analysis

Statistical analysis was performed using SPSS 15.0 for Windows (SPSS, Chicago, IL, USA). The Kolmogorov- Smirnov test was used to examine continuous variables with normal and abnormal distributions. One-way analysis of variance was used for normally distributed continuous variables and the Kruskal-Wallis test for abnormally distributed continuous variables. Nominal variables were analysed by Pearson's chi-square or Fisher's exact test, when applicable. Continuous variables are presented as mean-standard deviation (SD) or median (min–max), and categorical variables are presented as the number of cases and percentage. A P value of <0.05 was considered to be significant. Power analysis and sample size calculations were carried out using the G*Power 3.0.10 program (Franz Faul, Universität Kiel, Kiel, Germany).

## Results

From the 361 initial participants, 22 (6.09%) dropped out of the study. Therefore, 338 participants were included in the study: 175 in group 1 that used a soft catheter, 100 in group 2 that used a firm catheter and 63 in group 3 that used a tenaculum to ease the introduction (Fig . 1).

**Fig.1:**
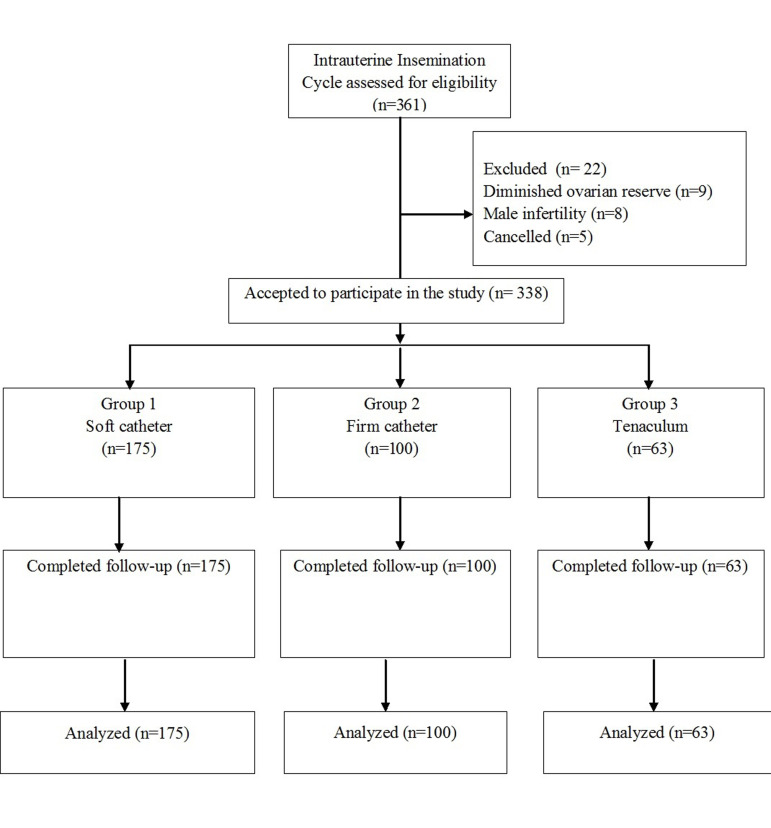
Enrollement and follow-up of the study subjects.

Table 1 lists the participants’ demographic characteristics and laboratory parameters. There were no significant differences between groups regarding age, body mass index (BMI), baseline hormone profiles, type and duration of infertility. Dose and type of gonadotropin (recFSH versus hMG), u-hCG versus rec-hCG utilization for trigger, luteal phase support, antral follicle count, number of follicles >17 mm and endometrial thickness on hCG day were comparable in all groups. Total progressive motile sperm count (TPMSC) and sperm morphology were also similar between the groups (P>0.05).

**Table 1:** Demographic characteristics and laboratory parameters of the patients


	Group 1 Soft (n=175)	Group 2 Firm (n=100)	Group 3 Tenaculum (n=63)	p

Age (Y)		26.53 ± 4.51	27.05 ± 4.98	27.97 ± 4.33	0.103^a^
BMI (kg/m^2^)		25.05 ± 3.46	24.66 ± 3.44	24.05 ± 3.19	0.135^a^
Primary infertility (%)		126 (72.0)	80 (80.0)	48 (76.2)	0.322
Secondary infertility (%)		49 (28.0)	20 (20.0)	15 (23.8)	
Duration of infertility (Y)		3 (1-12)	3 (1-14)	3 (1-16)	0.589^b^
Baseline FSH (IU/L)		6.78 ± 1.72	7.01 ± 1.88	6.44 ± 1.35	0.120^a^
Baseline LH (IU/L)		4.51 ± 1.56	4.77 ± 1.75	4.66 ± 1.85	0.454^a^
Baseline E2 (pg/mL)		41.21 ± 17.50	40.52 ± 14.04	41.19 ± 16.16	0.941^a^
Antral follicle count		10 (6-16)	10 (7-16)	10 (4-16)	0.115^b^
hMG(%)		85 (48.6)	45 (45.0)	23 (36.5)	0.165
rFSH (%)		90 (51.4)	55 (55.0)	40 (63.5)	
Duration of stimulation (D)		5 (5-16)	5 (5-13)	5 (5-16)	0.478^b^
rFSH dose (IU)		75 (37.5-225)	75 (37.5-187.5)	75 (37.5-112.5)	0.522^b^
hMG dose (IU)		112.5 (75-150)	150 (75-225)	75 (75-225)	0.251^b^
Number of cycle		2 (1-5)	2 (1-5)	2 (1-5)	0.723^b^
Number of >17 mm follicles		1 (1-3)	1 (1-4)	1 (1-3)	0.763^b^
Trigger	Pregnyl (%)	151 (86.3)	81 (81.0)	54 (85.7)	0.498
	Ovitrelle (%)	24 (13.7)	19 (19.0)	9 (14.3)	
E2 on hCG administration day (pg/mL)		398.54 ± 154.85	338.13 ± 15.01	537.92 ± 375.66	0.196^a^
TPMSC (x10^6^)		51.37 ± 22.17	53.35 ± 25.78	52.62 ± 25.57	0.801^a^
Morphology		6.94 ± 1.77	7.04 ± 1.60	6.98 ± 1.70	0.903^a^
Endometrial thickness on hCG day (mm)		8.94 ± 1.73	8.96 ± 1.72	9.02 ± 1.82	0.954^a^
Trilaminer sign (%)		159 (90.9)	94 (94.0)	56 (88.9)	0.476
Luteal phase support (%)		38 (21.7)	26 (26.0)	21 (33.3)	0.194


Data are presented as mean ± SD or n(%). SD; Standard deviation, ^a^; One-way ANOVA test, ^b^; Kruskal Wallis test, BMI; Body mass index, FSH; Follicle stimulan hormone, LH; Luteinizan hormone, E2: Estradiol, hMG; Human menopausal gonadotropine, hCG; Human corionic gonadotropine, and TPMSC; Total progressive motile sperm count. P<0.05 is statistical significant.

Table 2 summarizes the pregnancy related outcomes. There were 48 clinical pregnancies with a CPR of 14.2% (48/338) and the LBR per cycle was 11.53% (39/338), which was comparable to recent data (12). The CPR was significantly higher in the firm catheter (19%, 19/100) and tenaculum groups (31.7%, 20/63) compared to the group that used the soft catheter (5.1%,9/175) (P<0.001). Both the live birth/clinical pregnancy [84.2% (16/19), 80.0% (16/20), 77.8% (7/9); P=0.736] and miscarriage/clinical pregnancy [15.8% (3/19), 20.0% (4/20), 22.2% (2/9); P=0.736] were comparable in all groups (Fig . 2).

**Fig.2:**
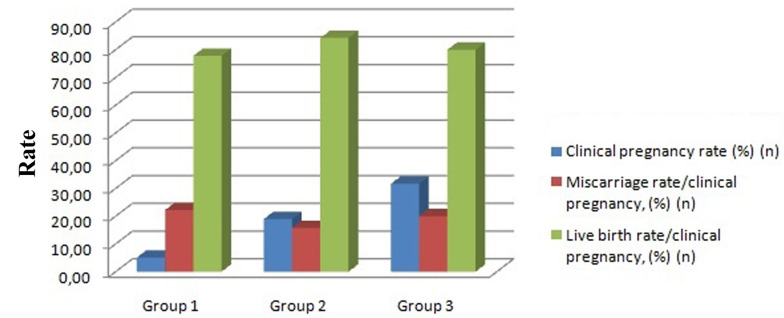
Perinatal outcomes of the groups. Group 1; Soft, Group 2; Firm, and Group 3; Tenaculum.

**Table 2:** Pregnancy related outcomes of soft, firm catheter and tenaculum applied patients undergoing IUI treatment


	Group 1 Soft (n=175)	Group 2 Firm (n=100)	Group 3 Tenaculum (n=63)	P value

Clinical pregnancy rate)	5.1 (9/175)^a, b^	19 (19/100)^a^	31.7 (20/63)^b^	<0.001^*^
Miscarriage rate/clinical pregnancy	22.2 (2/9)	15.8 (3/19)	20.0 (4/20)	0.736
Live birth rate/clinical pregnancy	77.8 (7/9)	84.2 (16/19)	80.0 (16/20)	


Data are presented as n (%). ^*^; Statistically significant, IUI; Intrauterine insemination, ^a^; Group 1 versus Group 2, and ^b^; Group 1 versus 3.

## Discussion

IUI is a commonly used cost-effective line of treatment for infertility (1, 13). In the literature, the pregnancy rate of IUI widely varies (e.g., 4-40%) (5, 6). This variation in pregnancy rates might be related to many factors, including the type of catheter used. The consistency of the ET catheter has been considered a determining factor in the success of ET procedures, whereas the impact of catheter type on IUI has been not been thoroughly investigated and limited data are available (10, 14).

In a study conducted by Smith et al., the pregnancy rates were not statistically different between the soft and firm catheter groups when a gentle technique was used and the technician did not touch the top of the fundus with the catether. (15). Lavie et al. observed by sonography that the firm catheters disrupted the three layer pattern of the endometrium in some patients who underwent IUI; however, they reported the same overall pregnancy rate with soft catheters (16). Similar outcomes were obtained in other related IUI studies (13, 17, 18). The results of a Cochrane data analysis indicated that there was no evidence of any significant difference between soft and firm catheters for IUI in terms of pregnancy related outcomes or adverse events (19).

Park et al. reported no significant differences in the CPR between non-using and using a tenaculum during intrauterine insemination (20). In contrast, Balci et al. suggested that uterine manipulation by applying a tenaculum to the cervix increased immediate uterine contractility and resulted in a higher pregnancy rate when they used ultrasound guidance to record the frequency of uterine contractions after insemination (12). Similarly, in our study, there was significantly greater CPR in the firm catheter and tenaculum groups compared to the soft catheter group. This difference in the success of the IUI and IVF treatments depended on the catheter type, and might be due to the difference between the location and timing of events during both procedures. In IUI, fertilisation takes place at the ampulla, away from the endometrium that is presumed to be damaged by a firm catheter. If any negative effect occurs in the uterine cavity during IUI, it may be achieved both by the volume of inseminated sperm and by the period of time until implantation, which is enough for natural recovery. Furthermore, in the course of artificial insemination, the uterine contractions induced by tenaculum application or by introduction of firm catheter might cause an immediate increase in passage of the sperm to the fallopian tubes, shorten the arrival time to the ampulla, and might disappear just before the fertilisation (14, 19).

On the other hand, endometrial scratch injury is a technique suggested by several studies to improve implantation rates in women who undergo in vitro fertilisation and have histories of recurrent implantation failure (RIF). Its application in IUI is less common. This procedure consists of applying a local endometrial travma to induce an acute inflammatory process and release of growth factors or proinflammatory cytokines, which arepresumed to improve decidualization and a subsequent successful implantation (21, 22). In a meta-analysis of 1871 IUI cycles, it was reported that endometrial scratch injury was associated with a higher CPR (OR 2.27) and ongoing pregnancy rate (OR 2.04) in comparison with the controls (11). Therefore, we suggest that inserting a firm catheter into the uterine cavity might have induced a local endometrial trauma and a subsequent inflammatory cascade, which resulted in a higher pregnancy rate compared to the gentle touch with the use of a soft catheter.

The limitations of this study are its retrospective design and small sample size. The primary aim of this study was to determine the difference in CPR between groups. According to the post hoc power calculation, ourgroup sample sizes of 175, 100 and 63 achievedan 80% power to detect a difference of 0.039 between the null hypothesis, which both group proportions were 0.124 and the alternative hypothesis that the proportion in the other group was 0.254 with a significance level of 0.05.

## Conclusion

This study showed that the application of a tenaculum or insertion of a firm catheter during the IUI might result in a higher CPR but does not alter LBR results. Further randomized prospective studies would be necessary to assess the long-term effects of catheter type and tenaculum use on IUI outcome.
